# Single port robotic hepatectomy: an initial European experience

**DOI:** 10.1007/s00464-026-12677-w

**Published:** 2026-04-06

**Authors:** P. A. Holzner, C. Berlin, S. Fichtner-Feigl, M. M. Menzel, B. Acidi, S. Caringi, M. Gelli, R. Memeo

**Affiliations:** 1 Department of General and Visceral Surgery, Center for Surgery, Medical Center and Faculty of Medicine, University of Freiburg, Hugstetterstraße 55, 79106 Freiburg, Germany; 2 German Cancer Consortium (DKTK) Partner Site, Freiburg, Germany; 3 IMMediate Advanced Clinician Scientist-Program, University of Freiburg, Freiburg, Germany; 4 Unit of Hepato-Biliary and Pancreatic Surgery, “F. Miulli” General Hospital, Acquaviva Delle Fonti, 70021 Bari, Italy; 5 Department of Medicine and Surgery, LUM University, Casamassima, 70010 Bari, Italy; 6 Department of Surgery, University of Rome “Tor Vergata”, Via Montpellier 1, 00133 Rome, Italy; 7 Department of Visceral and Oncological Surgery, Gustave Roussy, Cancer Campus, 94805 Villejuif Cedex, France

**Keywords:** Single port, Robotic, Hepatectomy, Liver resection, Feasibility

## Abstract

**Background:**

Single port (SP) robotic surgery is the latest innovation in robotic surgery. Published evidence is limited and few series are available for SP robotic hepatectomy. The aim of this study was to investigate early feasibility of SP robotic hepatectomy in Europe.

**Methods:**

Between February and June 2025, data on true SP and SP plus one robotic hepatectomies were systematically collected at three participating centres. The study focussed on the description of the cohort, feasibility, surgical outcome in terms of blood loss, postoperative complications according to Clavien–Dindo (C–D), specific posthepatectomy complications according to the International Study Group of Liver Surgery (ISGLS) definitions and length of hospital stay (LOS).

**Results:**

A total of 12 SP and SP plus one robotic hepatectomies were performed. The cohort comprises six females and six males with a median age of 62 (23.38–78.46) years and a body mass index of 25.9 (21.1–36.3) kg/m^2^. The series included seven non-anatomical/subsegment hepatectomies, two left lateral sectionectomies, two right hepatectomies and one segmentectomy. The median length of incision was 3.5 (2.7–4.7) cm plus a Pfannenstiel incision for larger specimen retrieval in two cases. An additional port (10 mm) was used in six cases. No procedure was converted to open, the median blood loss was 100 (0–580) ml. The median LOS was 3.5 days (1–8). According to the C–D classification, complications were categorized as follows: two grade I, two grade II and one grade IIIa complication. None of these met the criteria for ISGLS posthepatectomy complications. Follow-up at 30 days was uneventful in all cases.

**Conclusion:**

SP robotic hepatectomy is feasible when performed by experienced hepatobiliary surgeons. Larger series are needed to clearly define safety and the role of SP robotic hepatectomy within the armamentarium of hepatobiliary surgery and support the concept of tailored robotic-assisted surgery (T-RAS).

**Graphical abstract:**

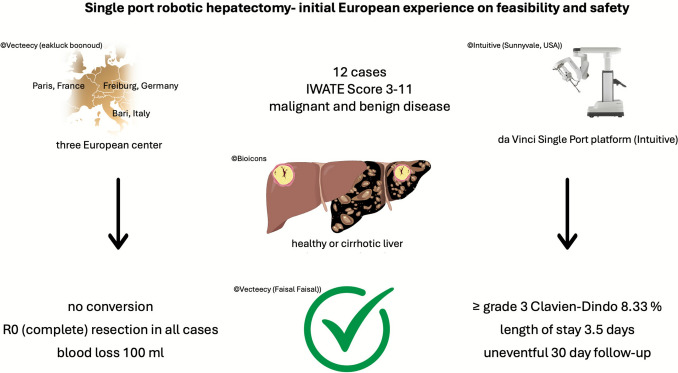

**Supplementary Information:**

The online version contains supplementary material available at 10.1007/s00464-026-12677-w.

Minimally invasive liver surgery continues to evolve with robotic platforms, increasingly expanding the possibilities of safe and complex hepatectomies. The advent of single port (SP) robotic surgery using the SP platform (Intuitive, Sunnyvale, USA) represents the latest innovation, allowing surgeons to perform intricate surgeries and hepatectomies through a single incision. This approach potentially minimizes abdominal wall trauma, reduces postoperative pain, expedites recovery, and improves cosmetic outcomes compared to conventional multiport (MP) techniques [1].

However, the clinical adoption of SP robotic hepatectomy in Europe has been limited to date, primarily due to regulatory approvals and technical constraints, including restricted instrument availability. Prior to its approval in Europe, only scarce clinical data on SP robotic hepatectomy existed, predominantly from Asian centres, where the SP platform had been introduced earlier. These preliminary reports, whilst encouraging, were limited [2–5]. Jang et al. from Korea recently reported the first series of 14 consecutive SP plus one robotic hepatectomies. Their series is the first published case series that includes major and minor hepatectomies [6]. There is even less data on true (without additional assistant port) SP robotic hepatectomy [7, 8]. Figure 1 demonstrates the setup for a true SP right hepatectomy at the centre in Freiburg.

**Fig. 1:**
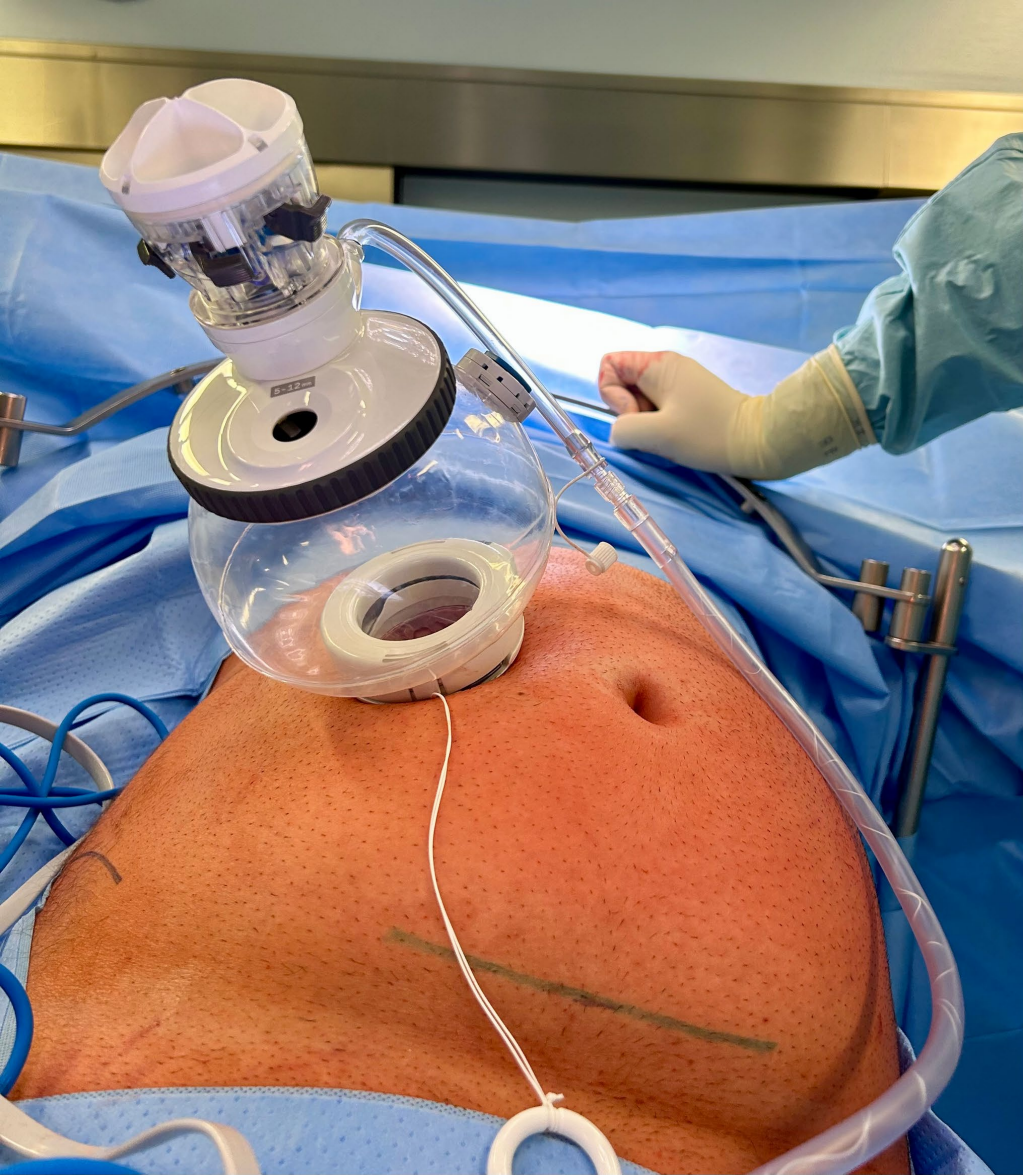
Illustrative setup for a true SP robotic right hepatectomy, showing the SP access in a right transrectal location

A major milestone for the European hepatobiliary surgery community occurred with the approval of the SP platform on January 24, 2024. This regulatory endorsement enabled European centres to initiate SP robotic programmes. To date, around 50 SP platforms are installed in Europe, with most centres focussing on non-hepatobiliary SP robotic surgery. Nevertheless, early adopters in all fields faced considerable challenges, such as the absence of dedicated irrigation/aspiration devices, limited stapling options, limited angulation and constraints in vessel-sealing instruments, which influenced patient selection and procedural complexity.

Given this pioneering context, rigorous evaluation of the feasibility of SP robotic hepatectomy during the initial implementation phase is essential. To standardize reporting on innovation in surgical robotics, the Idea, Development, Exploration Assessment and Long-term monitoring (IDEAL) framework encourages consideration of key stakeholders’ perspective at different stages during the life cycle of robot development and evaluation [9].

In this manuscript, we present a multicentre analysis of the initial European experience with SP robotic hepatectomy. Our focus is to systematically assess feasibility and surgical outcome. This study aims to establish early evidence to support the responsible expansion of SP robotic hepatectomy within the hepatobiliary surgical community.

## Material and methods

This retrospective, descriptive, multicentre feasibility study was conducted jointly at three tertiary referral centres: the Department of General and Visceral Surgery, University Medical Centre Freiburg, Germany; the Department of Visceral and Oncological Surgery, Gustave Roussy, Cancer Campus, Villejuif Cedex, France and the Hepatobiliary Surgery Unit, “F. Miulli” Hospital, Bari, Italy. The study was approved by the institutional ethics committee at Freiburg (No. 25-1322-S1-retro).

Data were prospectively collected and retrospectively analysed from consecutive patients scheduled to undergo SP robotic hepatectomy for benign or malignant liver lesions between February and June 2025 at the three participating centres. The use of an additional port (10 mm) and larger specimen retrieval via a Pfannenstiel incision was allowed, so the study includes true SP and SP plus one robotic hepatectomy. Each institution entered data into a standardized, secure electronic database using a uniform case report form to ensure consistency across sites. Recorded variables included patient demographics, comorbidities, American Society of Anaesthesiologists (ASA) classification, underlying liver disease, tumour characteristics, preoperative liver function, intraoperative details and postoperative outcome parameters.

### Patient preparation

All patients underwent standard preoperative work-up according to institutional protocols, including laboratory assessment of liver function, coagulation profile, cardiopulmonary evaluation, and cross-sectional imaging with contrast-enhanced CT and/or MRI to evaluate lesion characteristics and surgical suitability for SP robotic hepatectomy. Anaesthetic preparation was carried out according to local institutional standards and variably included arterial catheter, central venous lines, urinary catheter and occasionally epidural anaesthesia (Freiburg only).

### Main surgeon preparation

All procedures at each centre were performed by a single experienced MP robotic hepatobiliary surgeon had completed the MP robotic hepatectomy learning curve and reached the level of mastery as proposed by Kuemmerli et al. [10]. Furthermore, main surgeons underwent meticulous preparation and training on the SP platform. This included an individualized two-day dry/wet lab training at the Institut de Recherche Contre les Cancers de l'Appareil Digestif (IRCAD) in Strasbourg, a one-day dry lab training at the local Intuitive training centre (Freiburg only) as well as a self-guided simulator training (SimNow, Intuitive, Sunnyvale, USA). As the SP console is identical to the MP console, handling is straightforward to adopt. However, the platform itself, with its intracorporeally angulating elbows, necessitates a different strategy to overcome limitations in exposure and dissection. Two of the main surgeons performed smaller surgeries (e.g., hernia surgery, cholecystectomy, colorectal) prior to the first SP robotic hepatectomy. A multidisciplinary rehearsal on platform and patient positioning, required SP instruments, additional material (e.g., urinary catheter for Pringle manoeuvre) and emergency bailout strategies (bleeding, failure to progress) took place before the first case.

### Patient selection

Selection criteria for SP robotic hepatectomy differed slightly amongst the centres. Basically, tumours selected for the first case were located in easily accessible segments (segments 4, 5, left lateral section) but during the following cases even technically demanding hepatectomies were performed. Patients with a history of open surgery in the upper quadrants or the necessity of performing a lymphadenectomy or bilioenteric anastomosis were not selected at all centres. As there is no experience with patient selection in SP robotic hepatectomy in particular, selection otherwise was mainly adopted from MP hepatectomy protocols. A representative SP robotic hepatectomy video from each participating centre is provided as supplementary material (Supplementary Videos 1–3). The IWATE score for minimally invasive hepatectomies was used to calibrate the difficulty of each procedure [11].

### Postoperative management

Postoperative management at all centres followed early recovery principles, with an emphasis on mobilization, adequate analgesia, and close monitoring for complications, which were classified according to the Clavien–Dindo (C–D) grading system. Recorded complications were checked to meet criteria of specific posthepatectomy complications defined by the International Study Group of Liver Surgery (ISGLS). Investigated specific posthepatectomy complications are posthepatectomy liver failure (PHLF) [12], posthepatectomy bile leakage (PHBL) [13] and posthepatectomy haemorrhage (PHH) [14]. Outcomes assessed included length of hospital stay (LOS), blood loss, conversion rate and postoperative Visual Analogue Scale (VAS; scale from 0 to 10 indicating no pain to worst pain imaginable) at discharge.

### Analysis

Given the feasibility-focussed descriptive nature of the study, no formal hypothesis testing was performed. Continuous variables were summarized as medians with ranges, and categorical variables as counts.

## Results

Between February and June 2025, a total of 12 (four cases per centre) SP robotic hepatectomies were performed across the three participating centres. The median age at time of surgery was 62 (23.38–78.46) years with equal proportions of females and males. The body mass index (BMI) varied between 21.1 and 36.3 kg/m^2^ with a median of 25.9 kg/m^2^. Three patients presented with a known liver cirrhosis (Child–Pugh grade A). Staging of patients according to the ASA classification resulted in ASA grade I, ASA grade II and ASA grade III in one, four and seven patients, respectively (Table 1).

**Table 1:** Demographics, case characteristics and procedural details of 12 SP robotic hepatectomies

Case characteristics	
Patient characteristics	
Sex (*n*)	
Female	6
Male	6
Age [years; median, (range)]	62 (23.38–78.46)
BMI [kg/m^2^;(median, range)]	25.9 (21.1–36.3)
Cirrhosis (*n*)	3
ASA classification (*n*)	
ASA	1
ASA II	4
ASA III	7
Dignity of disease (*n*)	
Malignant	9
Benign	3
Indication (*n*)	
CRLM	5
LM (breast cancer, NET)	2
HCC	2
FNH	2
Suspicious gallbladder polyp	1
Procedural details	
Patient positioning (*n*)	
Supine	1
Left lateral decubitus	1
Port placement (*n*)	
Midline	9
Right transrectal	2
Right anterior axillary	1
Incision length [cm, median(range)]	3.50 (2.7–4.7)
Additional port (*n*)	6
Extent of surgery (*n*)	
Non-anatomical/subsegment	7
Right hepatectomy	2
Left lateral sectionectomy	2
Segmentectomy	1
Pringle manoeuvre (*n*)	6
SP instruments (*n*)	
Fenestrated bipolar forceps	12
Maryland bipolar forceps	12
Cadiere forceps	12
Clip applier	5
Monopolar curved scissors	4
Needle holder	4
Parenchymal transection (*n*)	
MAMBA	12
Use of stapler (*n*)	3
Suction method (*n*)	
Rigid device	8
Soft rubber catheter	4
Conversion to open (*n*)	0
Extraction site (*n*)	
Access port	10
Pfannenstiel	2
Total number of incisions	
True SP	5
True SP plus Pfannenstiel	1
SP plus one	5
SP plus one plus Pfannenstiel	1
Specimen margins (*n*)	
Negative margins; R0 (malignant)	9
Completely resected (benign)	3
IWATE Score (*n*)	
Score 3	2
Score 5	3
Score 6	3
Score 9	2
Score 11	2

*BMI* body mass index, *ASA* American Society of Anaesthesiologists, *CRLM* colorectal liver metastasis, *LM* liver metastasis, *NET* neuroendocrine tumour, *HCC* hepatocellular carcinoma, *FNH* focal nodular hyperplasia, *SP* single port, *MAMBA* moisture-assisted multiple bipolar, *IWATE* prefecture in Japan, where the 2nd International Consensus Conference on Laparoscopic Liver Resections (Morioka, Iwate) took place; *SP* single port

### Indication

Surgery was carried out for malignant and benign conditions in nine and three patients, respectively. Metachronous colorectal liver metastases (CRLM) were preoperatively diagnosed in five cases, liver metastases from non-CRC primary [breast cancer and neuroendocrine tumour (NET)], hepatocellular carcinoma (HCC) and growing focal nodular hyperplasia (FNH) in two patients each. One patient underwent SP robotic hepatectomy for a gallbladder polyp with suspicious wall thickening towards the liver (Table 1).

### Procedural details

**Fig. 2:**
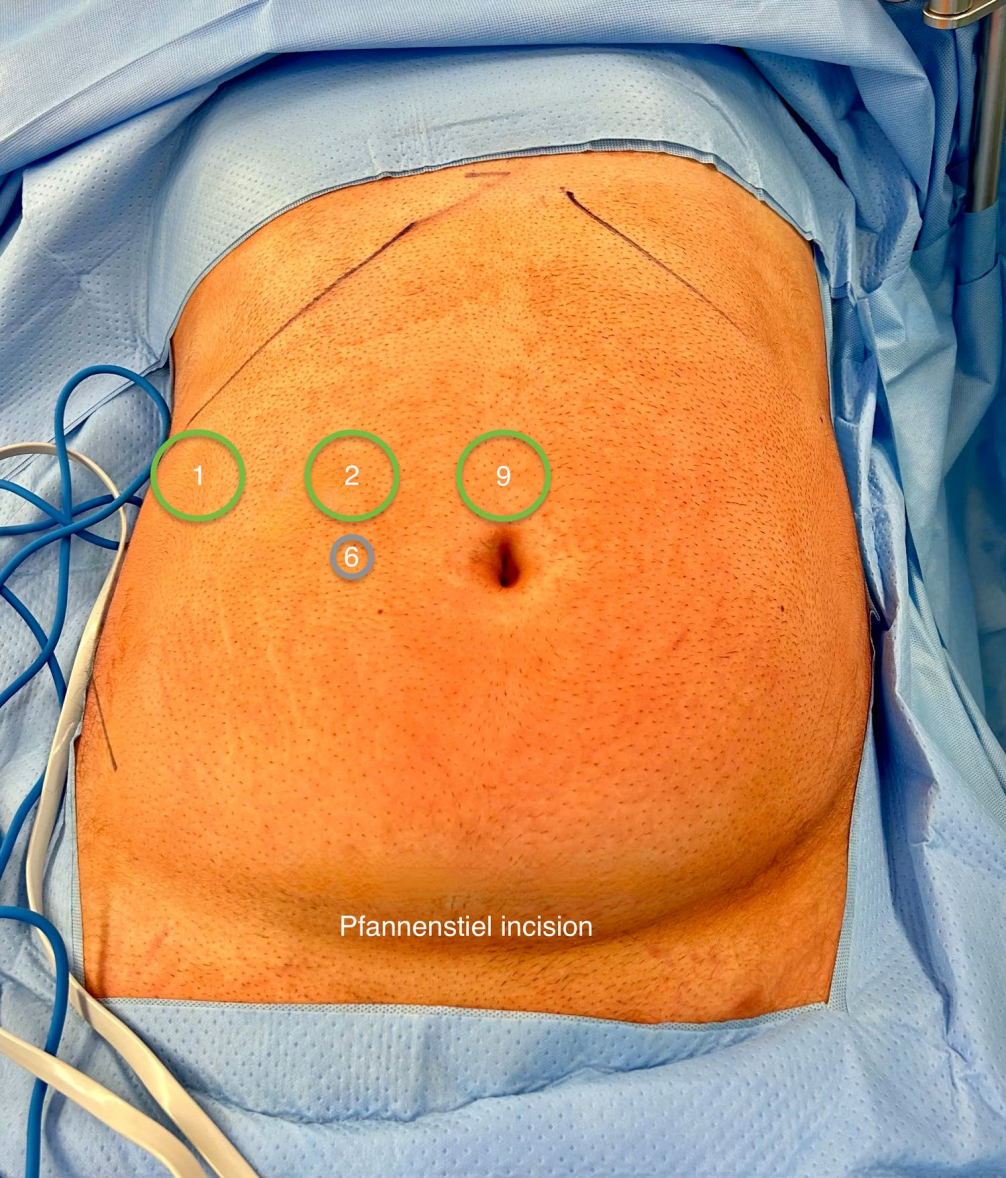
Setup for true single port (SP) and SP plus one hepatectomy in 12 cases. The access kit (green circle) was positioned either along the midline above the umbilicus (true SP and SP plus one), at the right transrectal site (true SP), or along the anterior axillary line (true SP). In the SP plus one configuration, an additional port (grey circle) was introduced to the right and caudal to the main access port. A Pfannenstiel incision was used for both true SP and SP plus one right hepatectomy procedures

Most patients were positioned supine with only one patient placed in a left lateral decubitus position. A midline incision was performed in nine cases. A right transrectal incision was used in two patients and an incision in the anterior axillary line was performed in one patient. The length of the incision ranged from 2.7 to 4.7 cm with a median of 3.5 cm. The policy regarding the use of an additional port varied between centres: The centre in Freiburg did not utilize additional ports in any case; the centre in Bari applied an additional port on demand in two cases; and the centre in Paris routinely employed an additional port in all cases. In total an additional port was used in six cases. In addition, in two cases (right hepatectomy) a Pfannenstiel incision was used for larger specimen retrieval. A schematic overview of access kit and additional port placement is given in Fig. 2. The present study includes seven non-anatomical/subsegment hepatectomies, one segmentectomy, two left lateral sectionectomies and two right hepatectomies (one with an additional left-sided non-anatomical hepatectomy). A Pringle manoeuvre was performed intracorporeally using an obliquely cut urinary catheter in six cases (Table 1).

**Fig. 3:**
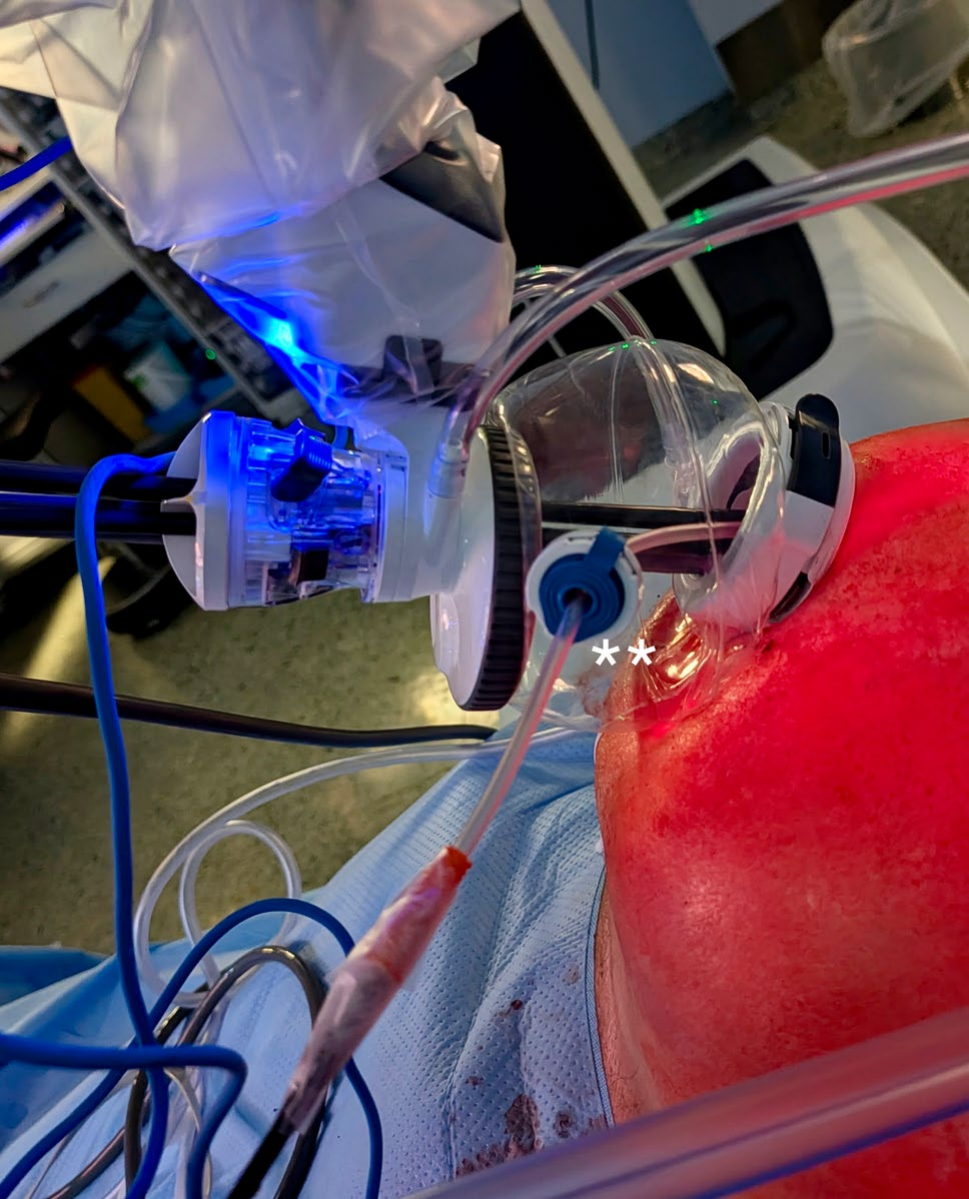
The soft rubber suction is introduced via the Chamber Seal (**) and carefully manoeuvered between the instruments and the camera, allowing continuous irrigation and aspiration. This setup is feasible even through small incisions, down to 2.7 cm, without interfering with instrument movement or visualization

The Fenestrated Bipolar, Maryland Bipolar and Cadiere Forceps were consistently used in all cases, whereas the Clip Applier was used in five and both the Monopolar Curved Scissors and the Needle Holder in four cases. Parenchymal transection was performed using the Moisture Assisted Multiple Bipolar (MAMBA) technique. The MAMBA technique is a fully robotic parenchymal transection technique, which is an adaptation of the clamp-crushing technique. It uses double bipolar instruments (Maryland and Fenestrated Forceps) for fine dissection, sealing and dividing of the parenchyma and small structures while the suction is used for intermittent irrigation to avoid sticky instruments [15]. A stapler fired by the table assistant was used in three cases. Irrigation and suction methods varied between centres. In eight cases (centres Bari, Paris), a rigid suction/irrigation system typically used in MP procedures was utilized via the access or additional port, whereas a soft rubber tube connected to a customized suction device was employed in the remaining four cases at the centre in Freiburg. The soft rubber suction device—introduced via the access port—was steered by the console surgeon but started by the table assistant on command, whereas the standard suction was routinely controlled by the table assistant (Fig. 3).

The IWATE score was calculated for all cases, with a median of 6 (3–11). No case required conversion to open surgery or conversion from SP or SP plus one to a modified MP approach with more than one assistant port. Specimen extraction in a retrieval bag for smaller hepatectomies was feasible through the access port itself in nine cases, occasionally after slight digital enlargement of the incision. Extraction of larger specimens (right hepatectomy) necessitated a Pfannenstiel incision in two patients. In malignant disease final histological examination revealed negative margins (R0) in all cases (Table 1).

### Surgical outcome

The median duration of surgery and console time were 247.5 (70–640) minutes and 217.5 (50–580) minutes, respectively. Reported blood loss ranged between 0 and 580 ml with a median of 100 ml. Postoperative outcome and complications according to the C–D classification are reported in Table 2. There was one C–D grade IIIa complication in a patient with liver cirrhosis who needed paracentesis to control ascites. Two patients were found to have C–D grade II complications, including one postoperative haematoma located within the hepatectomy defect without any further consequences or treatment and one postoperative small pulmonary embolism, requiring anticoagulation only. Neither the patient with ascites nor the patient with the haematoma met the ISGLS criteria for posthepatectomy liver failure (PHLF) and post hepatectomy haemorrhage (PHH), respectively. Furthermore, no posthepatectomy bile leakage (PHBL) occurred. Two patients with a drug-induced exanthema or an orthostatic dysregulation during early mobilization were graded C–D I. Postoperative analgesic regimens differed between centres. In eight patients at Bari and Paris postoperative non-opioid analgesics were administered as needed, whereas four patients at Freiburg were treated with non-opioids on a regular basis and oral opioids as needed. The VAS at the day of discharge did not differ between centres with five, six and one patient indicating VAS grade zero, VAS grade I and VAS grade III respectively. All patients tolerated the procedures well and were discharged between postoperative day one and eight, with a median LOS of 3.5 days. Recovery was uneventful in all cases at a 30-day follow-up.

**Table 2:** Results after a consecutive series of 12 SP robotic hepatectomies at three tertiary centres in Freiburg, Bari and Paris

Results	
Duration [min, median (range)]	
Total	247.5 (70–640)
Console	217.5 (50–580)
Blood loss [ml, median(range)]	100 (0–580)
Clavien–Dindo classification	
Grade I	2
Grade II	2
Grade IIIa	1
ISGLS complication	
PPH	0
PHBL	0
PHLF	0
Analgesics regimen	
Non-opioid as needed	8
Non-opioid regular basis	4
LOS [days, median(range)]	3.5 (1–8)
30-Day follow up	
Uneventful	12

No specific posthepatectomy complication occurred

*SP* single port, *ISGLS* International Study Group of Liver Surgery, *PPH* posthepatectomy haemorrhage, *PHBL* posthepatectomy bile leakage, *PHLF* posthepatectomy liver failure, *LOS* length of stay

## Discussion

Our multicentre series is the first in Europe to document SP robotic hepatectomy in routine clinical practice. Our findings suggest that SP robotic hepatectomy—either true SP or SP plus one—is feasible. We have demonstrated successful outcomes even for major and/or complex hepatectomies with zero conversion rates, little blood loss, and favourable postoperative outcomes.

A central point of debate concerns the use of an additional port during SP robotic hepatectomy. Amongst the three participating centres, strategies varied considerably, ranging from performing true SP hepatectomies to routinely applying an SP plus one approach, or reserving an auxiliary assistant port solely as a bailout option. Each strategy is legitimate and did not compromise outcome in our series, although true SP robotic hepatectomy may be slightly more time consuming than routinely using an additional port. Nevertheless, true SP robotic hepatectomy does not limit the extent of surgery or affect patient selection, as our series includes the first published true SP robotic right hepatectomy [7]. To date, there is no consensus on whether the use of an additional assistant port is allowed in SP robotic surgery. The border between SP plus one and a reduced port MP robotic approach is not defined. Therefore, further investigation and discussion are needed to determine whether SP plus one constitutes a reduced port MP approach or represents SP robotic surgery. Jang et al. also described the routine use of an additional port in their recently published relevant series on major and minor SP robotic hepatectomy [6]. Even though our current series comprises seven non-anatomical and subsegmental hepatectomies, we demonstrated that major hepatectomy is feasible even during early adoption when performed by experts.

Although the robotic approach may generally provide advantages, a definitive determination of superiority between the SP and MP platform is premature and arguably not meaningful to pursue [16]. Given the fundamental differences between these platforms, we anticipate that broader adoption of the SP robotic platform will enable more precise identification of optimal indications for each robotic platform. Importantly, the evolving concept of Tailored Robotic-Assisted Surgery (T-RAS) emphasizes selecting the most appropriate surgical approach for each individual patient and indication. This personalized strategy seeks to match the optimal robotic platform to the specifics of the lesion and patient anatomy, thereby maximizing surgical precision and outcomes whilst minimizing invasiveness. With larger series, growing experience and broader adoption of SP robotic hepatectomy, results should subsequently be compared with benchmark data on open and MP robotic surgery [17, 18].

Current data directly comparing SP robotic hepatectomy with MP robotic or laparoscopic (LS) hepatectomy remain scarce. A series by Na et al. found that SP robotic left lateral sectionectomy yielded significant improvements in LOS and blood loss, although operative times were longer [8]. Our experience supports these findings, with potentially longer operative times at least during the learning curve compared to MP hepatectomy or LS left lateral sectionectomy. In our series, the median blood loss was 100 ml (0–580 ml) across both minor and major hepatectomy, comparable to published series on LS or MP robotic hepatectomy [19]. The only comparable SP robotic hepatectomy series did not report on blood loss but described that one patient required blood transfusion. In contrast to our series, they allowed lymphadenectomy (three out of 14 cases) to be performed. Their operating time even with lymphadenectomy included (mean 201.1 min vs. mean 253.75 min; median 217.5 min) is shorter, whereas the LOS (mean 7.2 days vs. mean 4 days; median 3.5 days) is longer [6]. A possible explanation for the difference in duration of surgery is that our series includes six true SP robotic hepatectomies whereas Jang et al. routinely described a SP plus one approach. The series by Na et al. reported on comparable blood loss (mean 121.2 ml) after SP left lateral sectionectomies only [8].

**Fig. 4:**
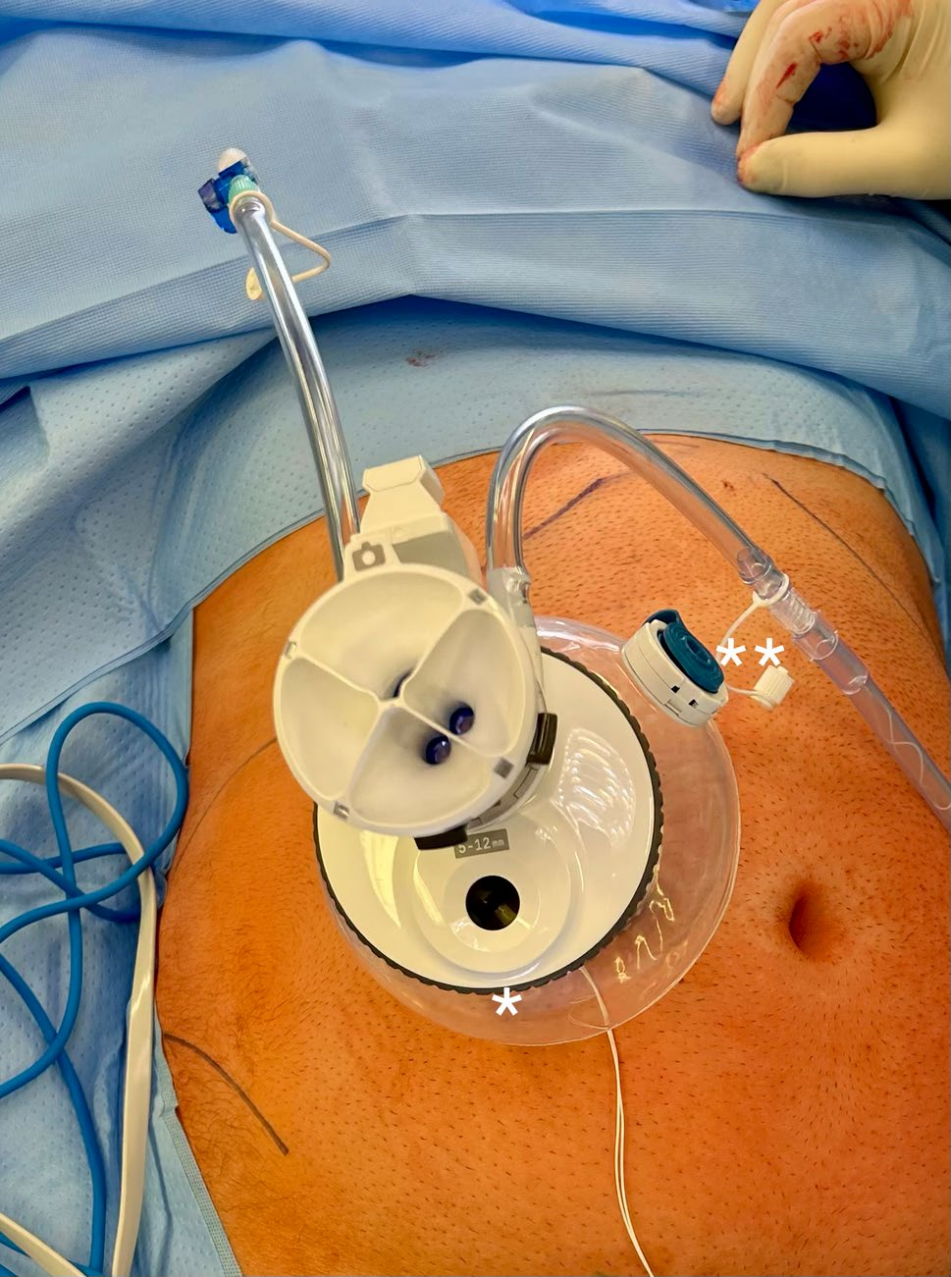
The SP access kit provides two additional seals: the larger Rotating Seal (*) and the Chamber Seal (**). In true SP hepatectomy, these seals facilitate the introduction of auxiliary instruments such as suction and staplers. The soft rubber suction is typically inserted through the Chamber Seal

The Brescia guideline on minimally invasive hepatectomy strongly recommends considering a Pringle manoeuvre to reduce blood loss—a technique also feasible during SP robotic hepatectomy using an obliquely cut urinary catheter fully intracorporeally [20]. In the present study, a Pringle manoeuvre was used in 6 of cases. In theory the access kit provides the opportunity to apply an extracorporeal Pringle by using the chamber or rotating seal. This supports the idea of using the extra seals to assist surgery without an additional port near the access kit (Fig. 4).

In our series, we observed an uneventful postoperative course in seven patients, reflecting aspiring early outcomes with no readmissions and an uneventful 30-day follow-up in all cases. One patient with cirrhosis developed symptomatic ascites requiring paracentesis (C–D grade IIIa) but was discharged uneventfully on postoperative day 8. A large study by Görgec et al. (Dutch Liver Collaborative Group) reported nationwide outcomes of hepatectomies, including 400 robotic MP hepatectomies, with a C–D grade ≥ III complication rate of 7%, which is within the range of our findings [21]. Li et al. established benchmark cutoffs for major complications after minor (≤ 5.2%) and major (≤ 16.7%) MP robotic hepatectomies which will be important for larger series. The series by Jang et al. described one PPH but otherwise no posthepatectomy specific or other major complication which supports our reported outcome [6]. With the same number of cases compared to our series but only left lateral sectionectomies Na et al. claimed that true SP robotic hepatectomy is both feasible and safe. Our findings support these findings in true SP or SP plus one hepatectomies, though larger series are needed to clearly demonstrate safety.

During our series, two thirds of patients required only non-opioid analgesics as needed, reflecting low postoperative pain after SP robotic hepatectomy before discharge. Evidence focussing on postoperative analgesic management after minimally invasive robotic hepatectomy is scarce compared to LS surgery. Joselyn et al. reported similar postoperative analgesic requirements after robotic versus LS gastrectomy in obese patients, with lower narcotic consumption in the robotic group [22]. A study by Lee et al. compared SP robotic with single incision LS surgery, showing equivalent postoperative analgesic consumption [23]. However, our series demonstrates a low VAS at discharge and a limited use of analgesics in general even during the hospital stay. With a median LOS of 3.5 days, we waived collecting or reporting the VAS at an earlier stage, and larger series are needed to investigate postoperative pain levels in more detail.

Regarding single incision laparoscopy, a systematic review by Benzing et al. reported shorter LOS and better cosmetic results compared to MP laparoscopy [24]. Our series supports extending this finding to SP over MP robotic hepatectomy, though larger studies are needed for confirmation. Especially true SP robotic hepatectomy harbours a potential cosmetic benefit over MP robotic hepatectomy. With a single incision—even larger than an eight mm incision in MP robotics—patient satisfaction could even be better. One study found that patient preference concerning cosmesis is a single incision compared to MP approaches. However, this study is limited because the single incision was made in the periumbilical fold [25]. Further investigation is needed to determine the threshold of the length of a preferable single incision and even the role of the additional port in SP plus one in this context must be explored. A study by Morgantini et al. demonstrated superiority of SP over MP patient-reported cosmetic and pain outcomes in urooncologic surgery [26]. From our point of view this could be of relevant interest for younger patients or females in general but not necessarily for obese patients because MP robotic hepatectomy also results in very low rates of surgical site infections. The drawback of a single, but longer (midline) incision during SP robotic hepatectomy could be a higher risk for incisional (port site) hernia. A possible adaptation to address this issue could be to avoid the midline and opt for a paramedian, transrectal access in cross-sectional technique whenever suitable.

The median LOS in our series was 3.5 days. Compared to the two available case series for SP robotic hepatectomy our LOS is slightly shorter. Na et al. with the series on true SP left lateral sectionectomy reported a LOS of a mean 5.2 days, compared to a mean 7.1 days reported by Jang et al. within their SP plus one series. The difference in LOS between both Asian series could be explained by the fact that Na et al. only reported on left lateral sectionectomy, compared to minor and major hepatectomies in the series by Jang et al. Our series demonstrates that an earlier discharge seems to be possible if surgery is performed by an experienced surgeon and postoperative treatment is embedded in an early recovery program. The fact that we had no readmission and an uneventful follow-up at 30 days supports this assumption. At Freiburg in Germany, national regulations mandated discharge only after completion of the minimum required LOS. As a result, patients remained hospitalized despite being clinically fit and personally ready for discharge. Thus, our reported LOS could have been even shorter and further discussion will arise as to whether same-day discharge surgery is an option for at least smaller resections.

Within the IDEAL logic, the current experience should be followed by coordinated efforts to: consolidate indications and procedural steps, contribute data to dedicated registries for robotic liver surgery, and design pragmatic comparative studies once technique and technology are sufficiently stable. In the longer term (IDEAL stage 4), robust follow-up and health-economic analyses will be necessary to determine oncologic durability, cost-effectiveness and the role of single port robotic hepatectomy in the overall treatment algorithm.

In summary, our results support early feasibility of SP robotic hepatectomy. The SP robotic platform potentially offers both surgical and patient advantages, though further research is required to clarify its safety and benefits particularly for complex hepatectomy, within the framework of Tailored Robotic-Assisted Surgery (T-RAS).

## Supplementary Information

Below is the link to the electronic supplementary material.

Supplementary file1 (MOV 43159 kb)

Supplementary file2 (MOV 168608 kb)

Supplementary file3 (MOV 328470 kb)

Supplementary file4 (DOCX 13 kb)
