# Supplementary material for: Single port robotic hepatectomy: an initial European experience
Source: Surg Endosc. 2026 Apr 6;40(7):5680–90. doi: 10.1007/s00464-026-12677-w (PMC13369734; doi:10.1007/s00464-026-12677-w)
Supplement: Supplementary file 4 — Supplementary file4 (DOCX 13 kb) [file 464_2026_12677_MOESM4_ESM.docx]

**Supplementary legends**

Supplementary video 1: This video demonstrates a single-port robotic non-anatomical segment 7 resection in a patient with a metastasized rectal cancer.

Supplementary video 2: This video demonstrates a single-port plus one resection of segment 4b with extension to segment 3

Supplementary video 3: This video demonstrates a single-port plus one robotic caudate lobectomy
